# Genome Sequence of Gordonia rubripertincta Phage Survivors, a Cluster CT Siphovirus

**DOI:** 10.1128/mra.01086-22

**Published:** 2023-01-04

**Authors:** Amber M. Amend, Jacob P. Bifone, Justin C. Brewer, Makayla N. Denton, Eden B. Gilbert, Abigail C. Grimm, Jaden M. Hogan, Reagan M. Kelley, Lennon J. Kelly-Brooner, Jit A. Mukerji, Matthew Osterhoudt, Cody R. Senn, Bethanie R. Smith, Olive G. Stillwell, Jimmy Vo, Danielle K. Watt, Pamela L. Connerly, Elizabeth E. Rueschhoff

**Affiliations:** a School of Natural Sciences, Indiana University Southeast, New Albany, Indiana, USA; Queens College Department of Biology

## Abstract

Bacteriophage Survivors is a siphovirus isolated from Gordonia rubripertincta NRRL B-16540. Survivors has a 45,436-bp genome encoding 69 predicted protein-coding genes, of which 32 have assigned functions. Based on gene content similarity to sequenced actinobacteriophages, Survivors is assigned to phage cluster CT.

## ANNOUNCEMENT

*Gordonia* bacteria are opportunistic human pathogens with varied, important roles in bioremediation (1). The characterization of *Gordonia* bacteriophages may allow for the development of additional biotechnology applications and phage therapy alternatives to antibiotics.

**FIG 1:**
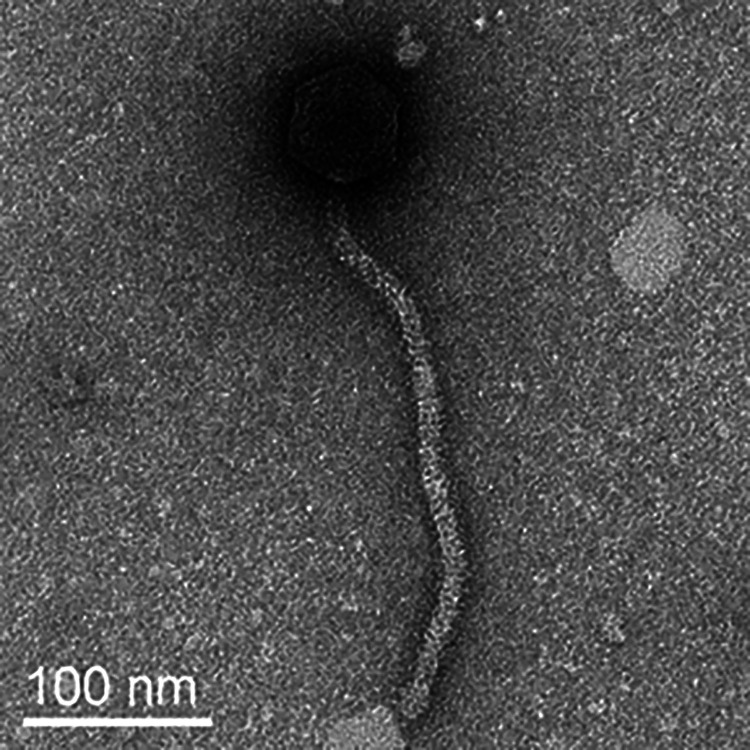
Negative-stain transmission electron microscopy of Survivors. TEM imaging reveals a Siphoviridae bacteriophage.

Bacteriophage Survivors was isolated from a soil sample from Jeffersonville, Indiana (Global Positioning System [GPS] coordinates 38.314609 N, 85.693118 W), using standard protocols from the Science Education Alliance-Phage Hunters Advancing Genomics and Evolutionary Science (SEA-PHAGES) Phage Discovery Guide (2). The soil sample was washed with peptone yeast calcium (PYCa) medium, and the filtered wash (0.02 μm pore size) was inoculated with Gordonia rubripertincta NRRL B-16540 and incubated at 26°C with shaking at 250 rpm. After 48 h, the mixture was filtered, and the filtrate was spotted onto a top agar lawn containing *G. rubripertincta*. Survivors was purified through three rounds of plating and formed clear plaques of 0.5 mm to 1 mm in diameter. Negative-stain transmission electron microscopy was performed at the Indiana University Bloomington Electron Microscopy Center. Five distinct particles of Survivors phage were imaged, showing *Siphoviridae* morphology (Fig. 1). The capsid diameter averaged 65.2 ± 4.8 nm, and the tail length averaged 284.2 ± 22.9 nm.

DNA was extracted using phenol-chloroform-isoamyl alcohol (25:24:1) (https://phagesdb.org/media/workflow/protocols/pdfs/PCI_SDS_DNA_Extraction_2.2013.pdf), prepared for sequencing using the New England BioLabs Ultra II library kit, and sequenced at the Pittsburgh Bacteriophage Institute using Illumina MiSeq (v3 reagents). There were 236,073 150-base single-end reads providing 735× coverage. The raw reads were assembled and checked for completeness using Newbler v2.9 and Consed v29, respectively, using default settings (3). The genome is 45,436 bp long with a GC content of 61.8% and 3′ single-stranded genome ends (5′-CGGTAGGCAT-3′). Genome termini were determined through similarity to known phages, an analysis of read start buildups, and coverage levels across the genome (3). Based on gene content similarity (GCS) of 35% or higher to phages in the Actinobacteriophage database (http://phagesDB.org), Survivors was assigned to cluster CT (4, 5) and shares the highest GCS to cluster CT phages Dre3, Gibbous, and Cleo (84.68%, 83.22%, and 81.99% GCS, respectively).

The genome was annotated utilizing the following programs: DNA Master (v5.23.6, build 2705 24 Oct 2021) (http://cobamide2.bio.pitt.edu), Glimmer (v3.02b) (6), GeneMark (v2.5p [09.08.06]) (7), Starterator (v.462; http://phages.wustl.edu/starterator/), Phamerator (Actino_Draft v462) (8), BLASTp (v2.13.0+) (9), HHPred (v57c87) (10), ARAGORN (v1.2.41) (11), tRNAscan-SE (v2.0) (12), TMHMM (v1.0.08) (13), and SOSUI (14). Default parameters were used for all programs. Sixty-nine genes were annotated as protein-coding genes. The functions of thirty-two gene products were predicted, including HNH endonuclease, thymidylate kinase, dUTPase, lysin B, and two proteins encoding domains of lysin A. Five gene products were predicted to be membrane proteins, and two genes were found with no homologs in the Actinobacteriophage database. No tRNAs were identified. The left arm of the genome (genes 1 to 30; transcribed rightward) encodes structure and assembly functions as well as lysin A, while the right arm of the genome (genes 31 to 52; transcribed leftward) encodes DNA metabolism functions as well as lysin B. In addition, gene *5* of Survivors occurs much earlier in the genome compared with homologs in other Actinobacteriophage genomes.

### Data availability.

Survivors GenBank accession no. is ON970576 and has been assigned Sequence Read Archive (SRA) no. SRX14485091.
